# A Study on Material Properties of Intermetallic Phases in a Multicomponent Hypereutectic Al-Si Alloy with the Use of Nanoindentation Testing

**DOI:** 10.3390/ma13245612

**Published:** 2020-12-09

**Authors:** Mirosław Tupaj, Antoni Władysław Orłowicz, Marek Mróz, Andrzej Trytek, Anna Janina Dolata, Andrzej Dziedzic

**Affiliations:** 1 Faculty of Mechanics and Technology, Rzeszow University of Technology, ul. Kwiatkowskiego, 37-450 Stalowa Wola, Poland; trytek@prz.edu.pl; 2 Faculty of Mechanical Engineering and Aeronautics, Rzeszow University of Technology, al. Powstańców 8, 35-959 Rzeszów, Poland; zois@prz.edu.pl (A.W.O.); mfmroz@prz.edu.pl (M.M.); 3 Faculty of Materials Engineering, Silesian University of Technology, ul. Krasińskiego 8, 40-019 Katowice, Poland; 4 Institute of Physics, University of Rzeszow, Pigonia 1, 35-310 Rzeszow, Poland; dziedzic@univ.rzeszow.pl

**Keywords:** hypereutectic Al-Si alloy, intermetallic phases, materials properties, nanoindentation tests

## Abstract

The paper concerns modeling the microstructure of a hypereutectic aluminum-silicon alloy developed by the authors with the purpose of application for automobile cylinder liners showing high resistance to abrasive wear at least equal to that of cast-iron liners. With the use of the nanoindentation method, material properties of intermetallic phases and matrix in a hypereutectic Al-Si alloy containing Mn, Cu, Cr, Ni, V, Fe, and Mg as additives were examined. The scanning electron microscope equipped with an adapter for chemical composition microanalysis was used to determine the chemical composition of intermetallics and of the alloy matrix. Intermetallic phases, such as Al(Fe,Mn,*M*)Si, Al(Cr,V,*M*)Si, AlFeSi, AlFeNi*M*, AlCuNi, Al_2_Cu, and Mg_2_Si, including those supersaturated with various alloying elements (*M*), were identified based on results of X-ray diffraction (XRD) tests and microanalysis of chemical composition carried out with the use of X-ray energy dispersive spectroscopy (EDS). Shapes of the phases included regular, irregular, or elongated polygons. On the disclosed intermetallic phases, silicon precipitations, the matrix, values of the indentation hardness (*H_IT_*), and the indentation modulus (*E_IT_*) were determined by performing nanoindentation tests with the use of a Nanoindentation Tester NHT (CSM Instruments) equipped with a Berkovich B-L 32 diamond indenter. The adopted maximum load value was 20 mN.

## 1. Introduction

The research on the application of aluminum-silicon alloys for manufacturing automobile engine cylinder liners are carried out mainly by industrial laboratories. For that reason, detailed reports on such studies are rarely published in generally available scientific literature. As a result, there are gaps in the knowledge concerning the forms of microstructure development in cylinder liner castings characterized, among other things, by high resistance to abrasive wear. It follows from the available technical literature that monoblocks and/or cylinder liners for some motorcar models of Porsche, Jaguar, Mercedes, and Audi brands were or currently are manufactured using hypereutectic Al-Si alloys with a silicon content in the range of 17–27 wt.% Si with the underlying technological processes patented under trade names such as ALUSIL^®^, LOKASIL I^®^, and LOKASIL II^®^ [1,2,3,4]. The LOKASIL concept (quasimonolithic concept) allows to obtain an appropriate microstructure in areas of extreme operating conditions and locally increase the functional properties of the engine block (i.e., wear resistance, thermal and dimensional stability). The composite inserts are obtained by the infiltration of highly porous, hollow cylindrical preforms made of ceramic fibres and silicon particles (LOKASIL I^®^) or only silicon particles (LOKASIL II^®^) with an aluminium alloy during the engine block casting process [1,2]. In turn, the ALUSIL^®^ technology based on the hypereutectic AlSi_17_Cu_4_Mg alloy were used to produce monolithic aluminium engine blocks. Primary Si particles precipitated during solidification, after machining and honing procedure, protect the cylinder bore surfaces and provide the expected tribological surface characteristics for proper engine operation [3,4]. Daimler-Benz have applied the SILITEC^TM^ process to apply silicon coatings in engine monoblocks made of hypereutectic aluminum-silicon alloy [5,6]. Another innovative engineering solution was implemented by BMW in a six-cylinder engine in which the block made of a magnesium alloy was used with cast-in cylinder liners of a hypereutectic aluminum-silicon alloy [7]. Research work was also carried out on the optimization of manufacturing engine monoblocks of hypoeutectic Al-Si gravitationally cast alloys, with the cylinder bearing surface made of a hypereutectic alloy. The cylinder liner surface was formed with the use of the TRIBOSIL^TM^ method. The process involved local melting of the surface with simultaneous feeding of a powder silicon-containing powder [8,9]. In the AL-CAST research concept developed by the company TRIMET Aluminium AG [10], traditional cylinder liners were replaced with new hybrid ones, the outer surfaces of which were made of near-eutectic aluminum-silicon alloy, whereas the inner surface was made of a hypereutectic Al-Si alloy containing about 30 wt.% silicon. On the other hand, authors of papers [11,12] conducted studies on the development of monolithic cylinder blocks of Al-20 wt.% Si alloy.

The present authors have also developed a hypereutectic Al-Si alloy and a geometrical structure for bearing surfaces of cylinder liners service properties of which were comparable to those of cast-iron liners [13]. One advantage of replacing cast iron with a lightweight Al-Si alloy is reduction of the overall mass of a car, resulting in a further reduction of fuel consumption. Modeling the microstructure of the alloy characterized by high resistance to abrasive wear and intended for operation in conditions of lubricated friction requires the establishment of relationships between chemical composition, morphology, and mechanical properties, such as the hardness and elastic modulus of intermetallics.

The effect of the morphology of intermetallic phases in aluminum-silicon alloys is usually considered from the point of view of its relationship with the decrease of resistance to loads of variable and dynamic nature. On the other hand, morphological properties of intermetallic phases depend on chemical composition of the alloy and conditions of its solidification.

The presence of plate-shaped β-Al_5_FeSi intermetallic phases is commonly considered undesirable in view of blocking the course of liquid material supply in the course of crystallization and the resulting deterioration properties of the alloy. It is known from the literature [14] that, with the increase of iron content from 0.57 wt.% to 0.97 wt.%, the shape of intermetallics evolves from that typical for phase β-Al_8_Mg_3_FeSi_6_, through Chinese scripture forms of β-Al_15_Fe_3_Si_2_, to plate-shaped β-Al_5_FeSi. Precipitations of phase β-Al_5_FeSi, in view of its shape, favor induction of stress concentrations and nucleation of cracks in structures operated in variable load conditions. According to Warmuzek et al. [15], the increase of iron content in Al-Si-Fe alloy from 0.5 wt.% to 3.0 wt.% can be connected with evolution of shapes of intermetallic phases from plate-like forms, through Chinese script-type features, to solid polyhedrons. Introduction of an addition of 0.5–1.5 wt.% manganese at slow cooling of Al-Si-Fe alloy is favorable for development of precipitations in the form of Chinese scripture, and in case of high cooling rates, even addition as small as 0.1 wt.% Mn is sufficient to trigger occurrence of precipitations of that shape. According to the same authors, the addition of manganese and chromium and increase of Al-Si-Fe alloy cooling rate allows to avoid occurrence of plate-shaped intermetallics. Also, Manasijevic et al. [16] noted that the addition of 0.25 wt.% manganese in AlSi13Cu4Ni2Mg alloy has an effect on development of plate-shaped intermetallic phases. In turn, the authors of reports [17,18] confirm that addition of Mn in LM13 alloy containing 1.2 wt.% Fe results in a change of β-AlFeSi phase volume share to the benefit of other intermetallics with more desirable morphologies. The authors of [19] examined a hypereutectic alloy Al-17.5 wt.% Si containing 1.2 wt.% Fe and found that that addition of 0.6 wt.% Mn resulted in evolution of morphology of phase β-AlFeSi into α-Al(Fe,Mn)Si which, as a consequence, resulted in an increase of resistance of the alloy to abrasive wear.

The authors of [20], by adding 0.67 wt.% Cr, were able to reduce the volume share of plate-shaped phase β-AlFeSi to the benefit of phase α-Al(Fe,Cr)Si with skeletal morphology. Timelli and Bonollo [21] demonstrated that addition of 0.15 wt.% Cr to AlSi_9_Cu_3_Fe alloy led to the development of a complex phase Al_x_(Fe,Mn,Cr)_y_Si_z_ with a polyhedral, star-like, and blocky shape. By increasing the Cr content from 0.057 to 0.15 wt.%, they achieved a relative increase in the alloy hardness (~8% HV). The effect of chromium on the change of β-AlFeSi phase morphology into phase α is also produced by vanadium. The authors of [22] added 0.5 wt.% V and 1.0 wt.% V to Al-20Si-5Fe alloy and found the presence of Al(Fe,V)Si phase with flower-like morphology. The occurrence of the phase Al_12_(Fe,V)_3_Si besides the phase Al_4.5_FeSi was found by authors of [23] in the alloy AlSi20/8009. According to reports [24,25], shapes of intermetallics rich in iron depend on manganese content. At 0.35–0.40 wt.% Mn, the intermetallic phase rich in iron is characterized by plate-like shape typical for β-Al_5_FeSi, while at higher content of magnesium, by precipitation forms typical for β-Al_8_Mg_3_FeSi_6_ phase [26]. According to the authors of [27], the change of nickel content has no effect on changes to the morphology of iron-based intermetallic phases. However, the authors of paper [28] claim that, at the Fe/Ni ratio value of 0.23 (in wt.%), phase Al_9_FeNi appears and forms a closed or semi-closed network resulting in an increase of mechanical strength properties of Al-12Si-4Cu-1Mg (in wt.%) alloy at temperature 350 °C. According to the results described by Murali et al. [29], the addition of beryllium in the amount of 0.2–0.3 wt.% to Al-Si alloy containing as much as 1.0 wt.% iron changes the morphology of phase β from needle-shaped into Chinese script-like forms. In turn, Stolarz et al. [30] showed that the plate-shaped β-Al_5_FeSi phase precipitations play a similar role in decreasing resistance to loads of variable nature as silicon precipitations.

Additions of Cu, Ni, Cr, and Mg are introduced to aluminum-silicon alloys, especially those used for engine pistons, in order to improve their mechanical properties and dimensional stability at elevated temperatures, mainly through the reinforcing effect of developing intermetallic phases [31,32,33,34,35,36]. The authors of [31] examined different variants of heat treatment for A356 alloy and found the presence of intermetallic phases with various morphologies: Mg_2_Si, AlFeSi, Al_12_Mg_2_Si, Al_8_Mg_3_FeSi_6_, Al_12_Si(Fe,Cr), Al_3_Cr, Al_8_Cr_5_, Al_9_Si, Al_12_Mg_17_, CrFe_4_, and AlCuMg. Further, the authors of the study [32], by adding Ni and Zr to Al-Si-Cu-Mg (A354) alloy, obtained phases Mg_2_Si, Al_2_Cu, Al_3_CuNi, Al_9_NiFe, Al_8_Mg_3_FeSi_6_, Al_5_Cu_2_, Mg_2_Si_6_, (Al,Si)_2_(Ti,Zr), and Al_3_Ni_2_ with needle-like, polygonal, and/or irregular shapes. They have found that one effect of adding 0.2 wt.% Zr and 0.2 wt.% Ni was the increase of strength properties of the alloy by about 30% at temperature of 300 °C. Some interesting results were obtained by authors of [33] who added copper in the amount of from 2.63 wt.% do 5.45 wt.% to the alloy Al-12.5Si-XCu-2Ni-1Mg (in wt.%) and found that with increasing content of the element, intermetallic phases rich in nickel, Al_3_Ni, and Al_3_CuNi transformed into the phase Al_7_Cu_4_Ni. Morphologies of the phases changed also from short-strip to reticular and then annular or semi-annular shape, respectively. That resulted in an improvement of mechanical properties of the alloy and an increase of its dimensional stability. On the other hand, the only intermetallic phases found in the alloy Al-12Si-4Cu-2Ni-2.6Mg (in wt.%) were *M*-Mg_2_Si, Al_7_Cu_4_Ni, and Al_5_Cu_2_Mg_8_Si_6_ [34]. In the two alloys, iron content was 0.19 wt.% Fe and 0.11 wt.% Fe, respectively. However, the authors of the above-quoted papers did not find presence of any intermetallic phases containing iron.

In turn, Li et al. [35] added 1 wt.% Ni and 0.1–3.2 wt.% Cu to an Al-Si alloy containing about 13 wt.% Si, about 1 wt.% Mg, and about 0.3 wt.% Fe. As a result, they observed presence of intermetallic phases Al_3_Ni, Al_3_CuNi, Al_7_Cu_4_Ni, Mg_2_Si, and Al_8_FeMg_3_Si_5_ and found that phase Al_3_Ni had a block-like morphology, phase Al_3_CuNi had the strip-like morphology, and phase Al_7_Cu_4_Ni had the skeleton-like morphology. Further, they proved that the phase Al_3_CuNi with strip-like morphology had the predominant effect on increase of strength of the alloy at elevated temperatures. In the next studies described in [36], the authors introduced 0.8 wt.% Fe and 0.5 wt.% Cr into the alloy Al-13Si-3.7Cu-3.2Ni-1.1Mg (in wt.%) and found presence of Cr, Fe, and Si in phase Al_3_CuNi which, by precipitating at boundaries of α-Al grains, might transfer stresses from the Al matrix. As a result of introducing Cr and Fe, a 26% rise of the tensile strength of the alloy was observed.

The presented review of literature reveals a wide range of opportunities to shape the morphology of intermetallic phases through the proper selection of both the chemistry of the alloy (introduction of alloying additives) and conditions of its crystallization. To be able to model microstructure of an alloy, it is necessary to understand the effect of chemical composition and conditions of crystallization on the microstructure and mechanical properties, such as the hardness and modulus of elasticity of individual phases. To ensure high resistance to abrasive wear, it is important that the knowledge on the subject is acquired with reference to all microstructure components of aluminum-silicon alloys.

## 2. Materials and Methods

### 2.1. Preparation of the Test Material

**Figure 1:**
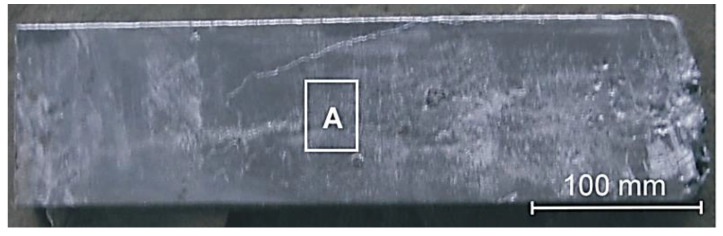
Plate casting with dimensions 400 mm × 120 mm × 40 mm cast in metal mold. A—test material taking area.

The aluminum-silicon alloy with chemical composition specified in Table 1 was prepared in an induction furnace with capacity of 5 kg. Modification with copper-phosphorus key metal was made at temperature 950 °C. The liquid metal at temperature of 920 °C was poured into a metal mould preheated up to 300 °C. The procedures for producing experimental castings are also described in [37,38]. From the plate casting with dimensions 400 mm × 120 mm × 40 mm, a specimen from area A (Figure 1) with dimension 40 mm × 30 mm × 10 mm was cut out using Labotom 3 metallographic cutter (Struers, Copenhagen, Denmark) equipped with Supra TRD 15 type water cooled cutting disc. The disc edge displacement linear speed was 37.2 m/s. The metallographic section was prepared on the specimen side with dimensions 40 mm × 30 mm. After sectioning, the surface was ground and polished with the use of Saphir 320E metallographic polisher equipped with Rubin 500 head (ATM, Altenkirchen, Germany). The finish polishing was made on a disc covered with suspension of silicon oxide with grain size of 0.1 μm.

### 2.2. Chemical Composition Microanalysis

**Figure 2:**
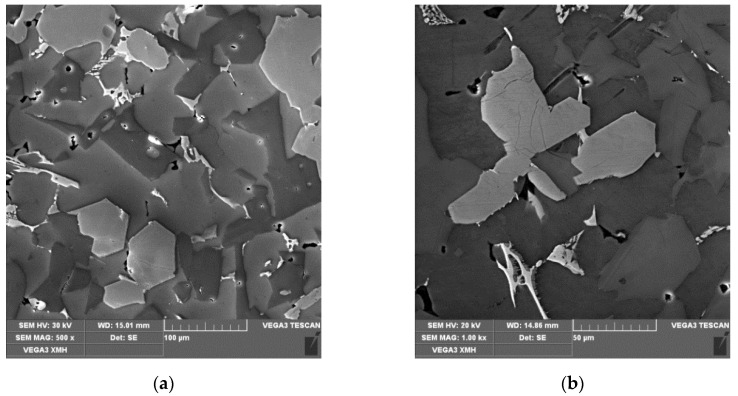
An example structure of hypereutectic Al-Si alloy prepared for metallographic examination, SEM, (**a**) 500×, (**b**) 1000×.

The analysis of chemical composition was carried out with the use of Q4 Tasman emission spectrometer (Bruker, Kalkar, Germany). The specimen was subject to examination with the use of VEGA XMH scanning electron microscope (SEM, Tescan, Brno, Czech Republic) equipped with an energy dispersive X-ray spectrometer (EDS, Oxford Instruments, Abingdon, UK) in order to reveal intermetallic phases (Figure 2). The determination of chemical composition was carried out for intermetallic phases and for the alloy matrix. The quoted results are averages from five measurements taken for each analyzed microstructure component.

### 2.3. Examination of Material Properties of Microstructure Components by Nanoindentation Testing

In the next step, the nanoindentation technique was used to determine the indentation hardness *H*_IT_ and the indentation modulus *E_IT_* evaluated with the use of Nanoindentation Tester NHT (CSM Instruments, Peseux, Switzerland). The measurements were taken in line with the standard PN-EN ISO 14577-1 [39]. The indentation hardness *H_IT_*, which is a measure of material resistance until permanent deformation, was determined from the following formula [39,40]:

(1) $$H_{IT}\;=\;\frac{F_{max}}{A_{p}}$$

where *H_IT_* (MPa) denotes the indentation hardness, *F_max_* (N)—maximum force applied to indenter, and *A_p_* (mm^2^)—area of the indenter-tested material contact surface projection.

The value of the indentation modulus *E_IT_* was determined from the formula [39,40]:

(2) $$E_{IT}\;=\;\;\frac{1-v_{s}^{2}}{\frac{1}{E_{r}}-\frac{1-v_{i}^{2}}{E_{i}}}$$

(3) $$E_{r}\;=\;\;\frac{\sqrt{\pi }}{2}\frac{S}{\sqrt{A_{p}}}$$

where *E_IT_* (GPa) is the indentation modulus; *ν_s_*—tested material Poisson number; *ν_i_*—indenter material Poisson number (for diamond indenter, *ν_i_* = 0.07); *E_r_* (GPa)—tested material reduced Young’s modulus; *E_i_*—elastic modulus of indenter material (for diamond indenter; *E_i_* = 1141 GPa); *S* = dF/dh—the slope of tangent to the load indentation curve during the unloading cycle; and *A_p_* (mm^2^)—area of the indenter-tested material contact surface projection.

**Figure 3:**
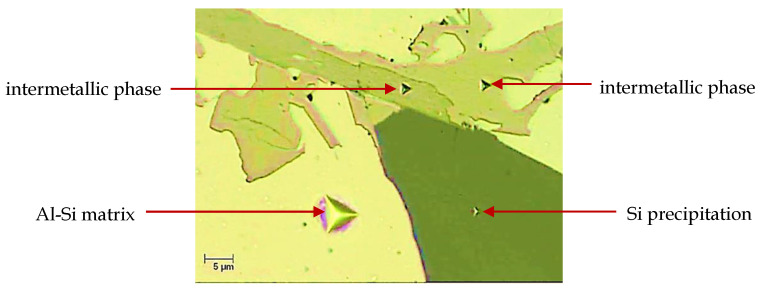
An example view of indents made in a silicon precipitation, in intermetallic phases, and in the matrix with the use of Nanoindentation Tester NHT (CSM Instruments).

Nanoindentation tests were carried out using B-L 32 diamond indenter. The maximum value of the load force was 20 mN. The indenter loading and unloading rate for all the analyzed microstructure components was 40 mN/min. The maximum load force interaction period was 15 s. Measurements were taken for silicon precipitations, intermetallic phases, and the matrix. In the case of intermetallics and silicon precipitations, measurements were taken on precipitations with largest dimensions to minimize the effect of immersion in matrix in the course of measurement which results in underestimation of values of the analyzed material properties. Example views of indents made on a silicon precipitation, intermetallics, and the matrix are shown in Figure 3.

Results of material properties (nanoindentation) tests are presented in the form of variability ranges for five measurements.

### 2.4. X-ray Diffraction

The phase composition analysis was performed using a Bruker D8 Advance X-ray diffractometer with the Cu lamp (Kα1 = 1.5406 Å). To solve the resulting diffractograms, a base of ICCD PDF standards was used. Angle and spot intensity matching were analyzed using the EVA software.

## 3. Results and Discussion

### 3.1. Identification of Alloy Microstructure Components

**Figure 4:**
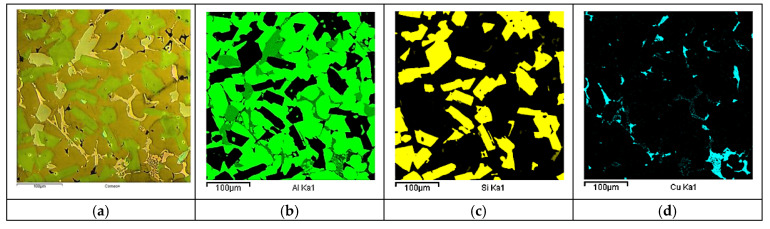

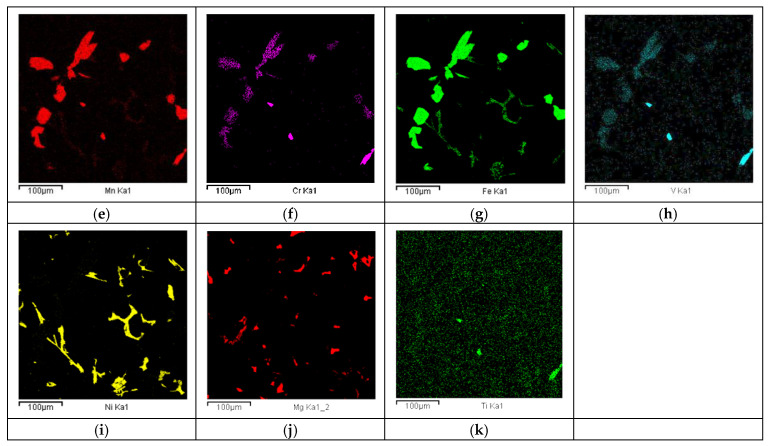
SEM micrograph, CAMEO image (**a**) and mapping of elemental distribution (**b**–**k**) of hypereutectic aluminum-silicon alloy.

**Figure 5:**
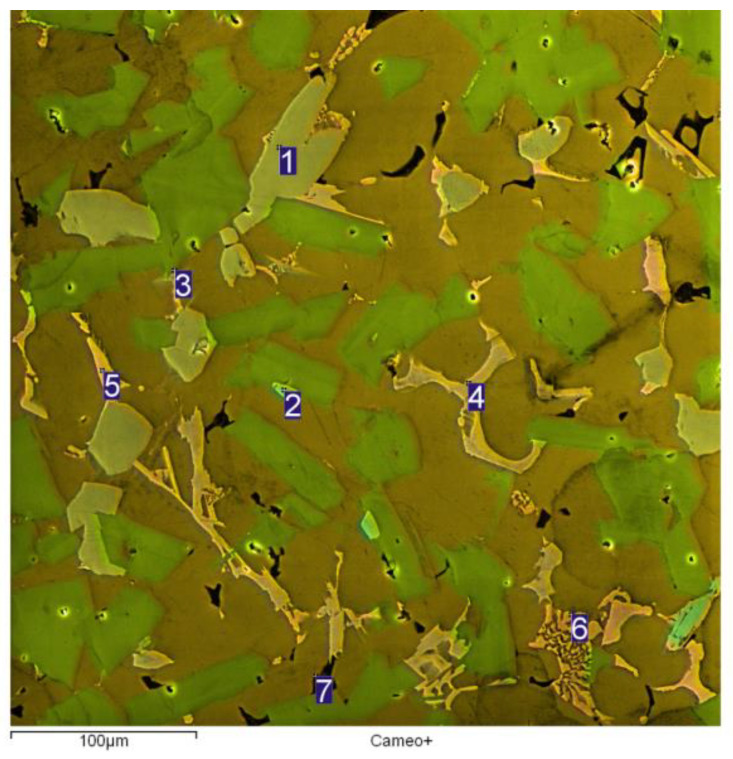
Microstructure of hypereutectic aluminum-silicon alloy with the characteristic microstructural components of various chemical composition, SEM, secondary electrons (SE).

**Figure 6:**
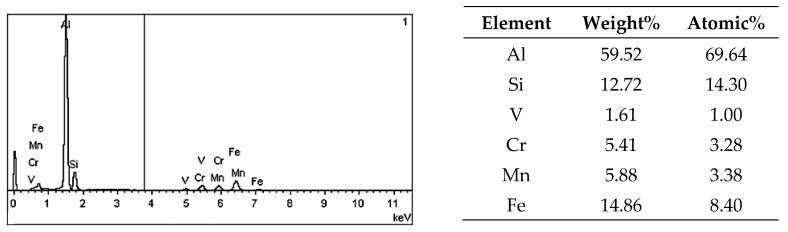
EDS diagram and chemical composition at point no. 1 of Figure 5.

**Figure 7:**
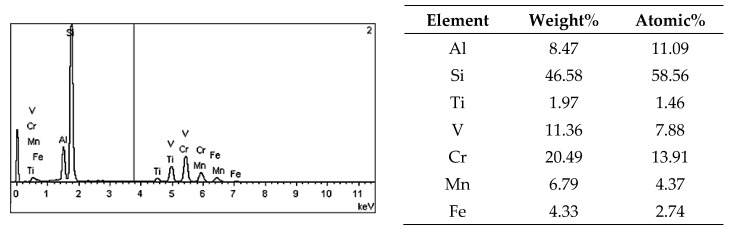
EDS diagram and chemical composition at point no. 2 of Figure 5.

**Figure 8:**
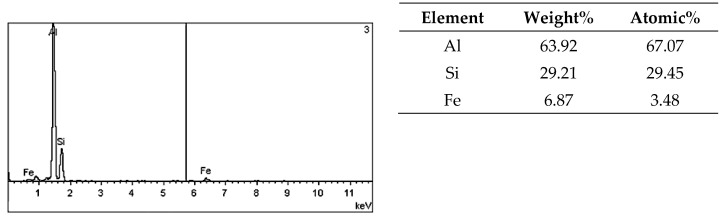
EDS diagram and chemical composition at point no. 3 of Figure 5.

**Figure 9:**
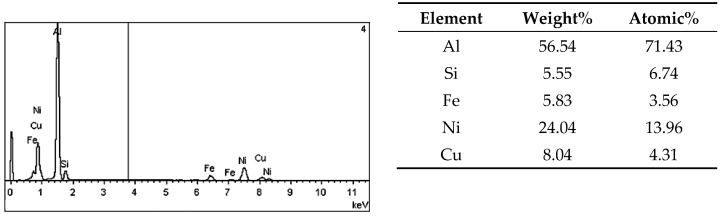
EDS diagram and chemical composition at point no. 4 of Figure 5.

**Figure 10:**
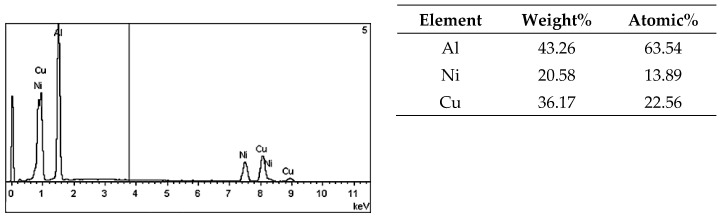
EDS diagram and chemical composition at point no. 5 of Figure 5.

**Figure 11:**
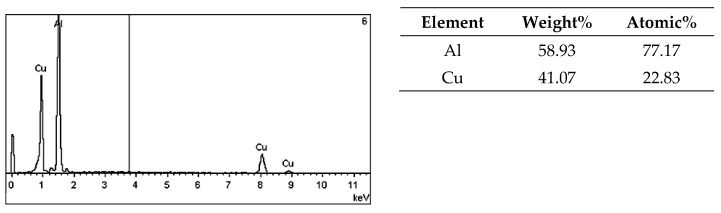
EDS diagram and chemical composition at point no. 6 of Figure 5.

**Figure 12:**
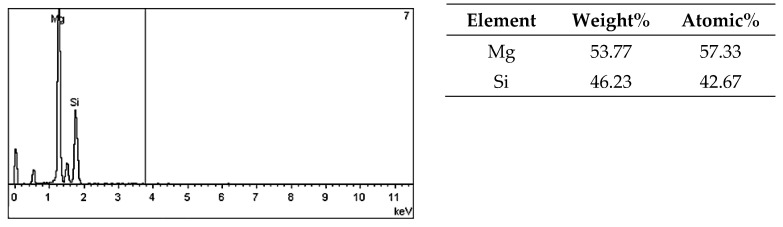
EDS diagram and chemical composition at point no. 7 of Figure 5.

**Figure 13:**
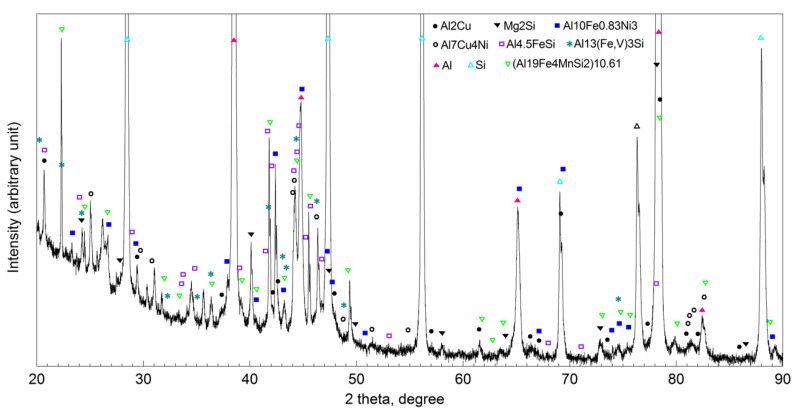
XRD patterns of Al-30 wt.% Si with Mn, Cu, Cr, Ni, V, Fe and Mg additions.

On the metallographic section surface, distribution of alloying elements was mapped results of which are shown in Figure 4. To determine chemical composition of microstructural components to necessary accuracy, point analysis was carried out (Figure 5). Example results of EDS diagrams and quantitative point analysis of chemical composition for individual microstructure components of the alloys are presented in Figure 6, Figure 7, Figure 8, Figure 9, Figure 10, Figure 11 and Figure 12. To identify intermetallic phases in the examined alloy, the X-ray diffraction (XRD) method was also used. Results of X-ray phase analysis are shown in Figure 13.

The authors of papers [22,41,42] proved that, for intermetallic phases in which the share in the alloy was less than 5 vol.%, the sensitivity of the X-ray diffraction (XRD) method was too low to detect and identify them. Still, another approach was adopted by the authors of [43] who marked some of peaks on diffraction patterns as unknown, which may be evidence of the unavailability of databases concerning multicomponent intermetallic phases. By analyzing the cooling curves and their derivatives for a AlSi_9_Cu_3_Fe alloy containing additions of Cr, Mo, V, and W, the authors of the paper [44] found the presence of a quadruple eutectic, one component of which was the phase AlSiCuFeMgMnNi*X*, with *X* denoting alloying elements (Cr, Mo, V, W) or their combinations. This evidences that a multicomponent silumin may comprise complex intermetallic phases which cannot be identified correctly with the use of the X-ray diffraction (XRD) method. Elements with similar values of atomic radii may change both morphology and stoichiometry of intermetallic phases [45]. That is the reason why many authors of research papers dealing with identification of intermetallics composed of more than three elements refrain from quoting complete stoichiometry of the phases [21,46,47]. Such an approach is also adopted in the present work.

The phase analysis was carried out with the use of results of both EDS (Figure 6, Figure 7, Figure 8, Figure 9, Figure 10, Figure 11 and Figure 12) and XRD (Figure 13). Based on results of the tests, the following were identified: (1) an intermetallic phase Al(Fe,Mn,*M*)Si with the shape of large polygons, often merged with each other, supersaturated with chromium and vanadium (*M* = Cr, V); (2) an intermetallic phase Al(Cr,V,*M*)Si, polygonal in shape, supersaturated with manganese, iron, and titanium (*M* = Mn, Fe, Ti); (3) Si crystals; (4) an intermetallic phase Al_4.5_FeSi with the shape of elongated polygons; (5) intermetallic phase AlFeNi*M* in the form of elongated polygons, supersaturated with copper and silicon (*M* = Cu, Si), (6) an intermetallic phase Al_7_Cu_4_Ni with the shape of irregular elongated polygons; (7) an intermetallic phase Al_2_Cu in the form of irregular elongated polygons; and (8) an intermetallic phase Mg_2_Si having the shape of irregular polygons.

In turn, the chemical composition of the matrix varied in the range of 96.82–97.89 wt.% Al, 1.02–1.73 wt.% Cu, 0.97–1.41 wt.% Si, 0.0–0.44 wt.% Mg, and 0.0–0.12 wt.% Ni.

The authors of papers [48,49,50] underline the favorable effect of alloying elements dissolved in matrix on its reinforcement. That is important in view of development of deformation and then microcracks on boundaries between the matrix and hard and brittle silicon crystals or intermetallics as a result of existence of loads with variable directions and values. The issue is widely analyzed with reference to the nucleation and development of fatigue cracks in silumins [30]. Therefore, enrichment of the matrix in silicon, copper, and magnesium demonstrated in the course of tests should result in a decrease of its susceptibility to the nucleation of cracks on the matrix-intermetallics boundaries as well as in a decrease of susceptibility to the propagation of cracks through the matrix between precipitations.

The obtained research results indicate that the newly developed chemical composition of the strongly hypereutectic silumin for cylinder liners [13] and the applied conditions of cooling enabled the crystallization of intermetallic phases with complex chemical compositions. The applied cooling rate prevented crystallization of large primary silicon precipitates and intermetallics which is favorable in view of variable stress states which occur in engine cylinder liners in the course of operation. Another significant circumstance is also the enrichment of the silumin matrix with alloying elements, which results in its reinforcement.

### 3.2. The Nanoindentation Test

**Figure 14:**
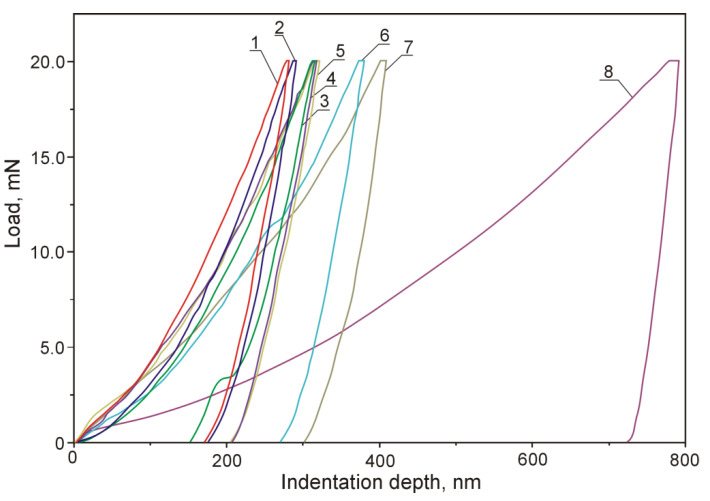
Plots representing indenter face displacement in the course of loading and unloading in the course of indentation tests of: (1)—Al(Fe,Mn,*M*)Si; (2)—Al(Cr,V,*M*)Si; (3)—Si crystals; (4)—Al_4.5_FeSi; (5)—AlFeNi*M* (6)—Al_7_Cu_4_Ni; (7)—Al_2_Cu; and (8)—the matrix.

**Figure 15:**
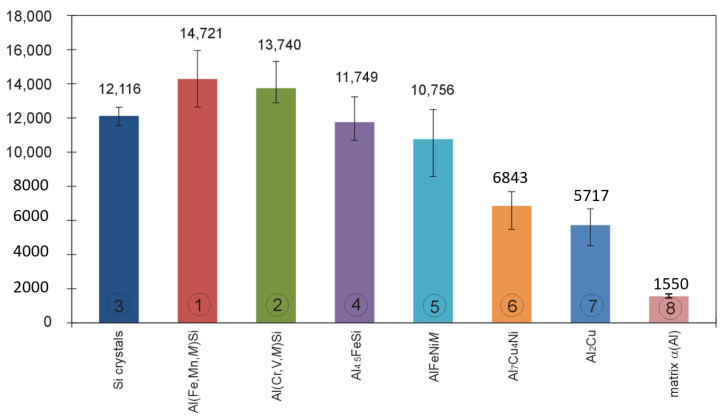
Values of indentation hardness *H_IT_* for primary silicon crystals, intermetallic phase crystals, and matrix of hypereutectic silumin determined with the use of indentation technique.

**Figure 16:**
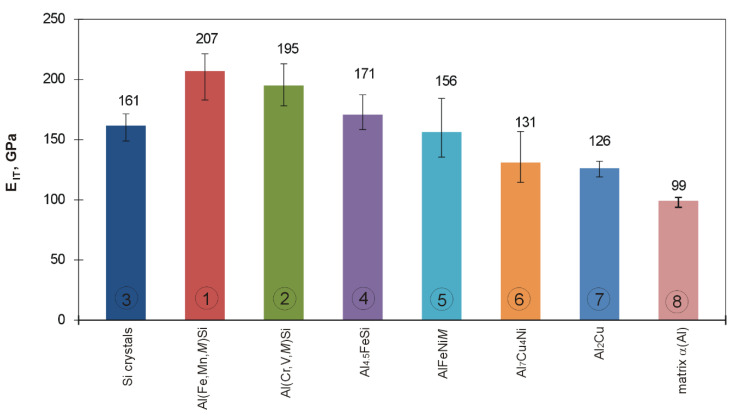
Values of indentation modulus *E*_IT_ for primary silicon crystals, intermetallic phase crystals, and matrix of hypereutectic silumin determined with the use of indentation technique.

Displacement of the indenter face in the course of indentation testing individual microstructure components of the examined silumin is shown in plots of Figure 14, whereas the bar charts of Figure 15 and Figure 16 represent values of hardness *H_IT_* and modulus *E_IT_* for the examined microstructure components of hypereutectic (Al-30% Si) aluminum-silicon alloy. Nanoindentation tests were not carried out for the phase Mg_2_Si because of small dimensions of its precipitations and the indenter load value (20 mN) adopted throughout the study.

Nanoindentation tests were carried out on unetched specimens in order to minimize plastic response of matrix material to the load applied to crystals.

The indentation test with the use of a Berkovich indenter revealed a difference in the course of plot lines representing indenter loading and unloading in the case of individual microstructure components. The difference was caused by the elastic and plastic response of the material constituting the analyzed microstructure features.

In case of the Al(Fe,Mn,*M*)Si intermetallic phase (1), the steepest course of the loading-unloading curve was observed which evidences that *H_IT_* and *E_IT_* assume highest values within the group of analyzed microstructure components. It can be found interesting that two intermetallic phases (1,2) were characterized by high values of hardness *H*_IT_ and modulus *E_IT_* compared to those demonstrated by silicon precipitations. In case of the Al(Fe,Mn,*M*)Si intermetallic phase (1), the indenter penetration depth was 282 nm. On the other hand, in case of the Al(Cr,V,*M*)Si intermetallic phase (2), the indenter penetration depth was 291 nm. In the case of phase (1) Al(Fe,Mn,*M*)Si, *H_IT_* value was included in the range 15,947–12,637 MPa, whereas the range of *E_IT_* values was 222–183 GPa. The intermetallic phase (2) Al(Cr,V,*M*)Si was characterized with *H_IT_* values falling into the range 15,306–12,887 MPa and *E_IT_* values in the range 213–178 GPa. The authors of [22] added 0.5 wt.% V and 1.0 wt.% V to the alloy Al-20Si-5Fe (in wt.%) and found that vanadium atoms can partially or entirely substituted the iron in the phase Al_4_FeSi_2_ forming thus the phase Al_4_(Fe,V)Si characterized with lower free energy values. The effect was a reduction of the Young’s modulus value for the alloy. Lower *E_IT_* values for the phase (2) Al(Cr,V,*M*)Si compared to the phase (1) Al(Fe,Mn,*M*)Si can be attributed to high content of vanadium in the phase (2).

In the case of primary silicon crystals (3), the indenter penetration depth was 312 nm. The unloading portion of the penetration graph features a prominent drop. This occurrence can be explained by metastable reconstruction of its crystalline structure and development of amorphous allotropic form in the course of deformation under indenter pressure [50,51]. According to Hu et al. [52], such reconstruction requires the pressure value of 11.2–12.0 GPa. As a result of lattice reconstruction, phase Si-I transforms into phase Si-II crystalline form which is accompanied by density increase by 22% [53]. The authors of [54] evaluated the effect of the load applied to the indenter on the indentation hardness value of silicon precipitations. The nanoindentation testing was carried out with the use of Vickers indenter and load force values in the range 200–800 mN. It was found that, with an increasing load force value, the hardness value decreases. The authors explain the effect by occurrence of excessive plastic deformations in the matrix in the form of pile-ups around the examined precipitation and by an increase of silicon precipitation volume due to interaction with the indenter. That resulted in the development of cracks in subsurface areas of silicon precipitations which, by merging with each other, were the cause of crumbling silicone particles in the area around the indenter.

Results of indentation hardness and indentation modulus measurements indicate that primary silicon crystals were characterized by an *H_IT_* value in the range 12,626–11,579 MPa and *E_IT_* value in the range 171–148 GPa. The results can be regarded as comparable with results reported by authors of [55] who observed values of indentation hardness *H_IT_* in the range from 11,500–12,500 MPa and *E_IT_* values in the range from 160 GPa to 165 GPa.

In the case of the Al_4.5_FeSi intermetallic phase (4), the indenter penetration depth was 316 nm. Values of *H_IT_* were included in the range 13,234–10,691 MPa and the range of *E_IT_* values was 188–159 GPa. Crystals of that intermetallic phase were characterized by indentation hardness *H_IT_* values close to those obtained for primary silicon crystals. However, in that case, the obtained values of the indentation modulus *E_IT_* were higher compared to values observed for primary silicon crystals, the fact being observed also in [55], where the same load force of 20 mN was adopted. The indenter loading and unloading rates were not quoted. Values of *H_IT_* are directly correlated with the indenter penetration depth (indenter loading curve in Figure 7), whereas *E_IT_* values are correlated with elastic response of the material (unloading curve in Figure 7). The analysis of the loading-unloading plot for the silicone precipitation reveals a notch on the unloading curve connected with metastable restructuring of the crystal lattice [50,51,52,53]. This can be considered a rationale behind lower *E_IT_* values observed for silicon crystals compared to intermetallic phases (1) Al(Fe,Mn,*M*)Si, (2) Al(Cr,V,*M*)Si, and (4) Al_4.5_FeSi.

The literature on the subject describes attempts to establish a relationship between the temperature of crystallization and the enthalpy and material properties of intermetallics. The authors of [56] have found that, in the case of multicomponent silumins, presence of iron, manganese, cobalt, chromium, molybdenum, and tungsten favor the crystallization of intermetallic phases at temperatures higher than the α+β eutectic crystallization temperature, whereas nickel, copper, and magnesium support the crystallization of intermetallics at temperatures lower than the eutectic crystallization temperature. Vanadium and chromium are linked to the reduction of size of the needle-shaped phase β-AlFeSi precipitations [57] and a decrease of its volume fraction at expense of α–Al(Fe,*M*)Si phase precipitations in the form of Chinese script or polygons [58]. According to Lin et al. [58], an addition of 0.8 wt.% V to the alloy A356 with elevated content of Fe (about 1.5 wt.%) resulted in reduction of volume fraction of the phase β-Al_5_FeSi through its partial replacement with precipitations of the phase α–Al_15_(Fe,V)_3_Si_2_ with Chinese script-like morphology. According to Yang et al. [20], an addition of 0.67 wt.% Cr to the alloy Al-12.5Si-5Cu-2Ni-0.8Mg-0.5Fe (in wt.%) results in the replacement of the needle-shaped phase β-Al_5_FeSi with the fishbone-shaped phase α–Al(Fe,Cr)Si. The authors of [59] explain the phenomenon by higher temperature of crystallization of the phase α–Al(Fe,*M*)Si which, by bonding Fe, hinders the development of the needle-like phase β-AlFeSi crystallizing at lower temperatures. The higher crystallization temperature requires higher enthalpy which is connected with higher value of *E_TI_* [55].

For the intermetallic phase (5) AlFeNi*M*, the indenter penetration depth was 320 nm, while for the intermetallic phase (6) Al_7_Cu_4_Ni, the depth increased to as much as 380 nm. From among all the examined crystals, the most gentle course of the loading-unloading plot line was observed for crystals of the intermetallic phase (7) Al_2_Cu. In that case, the indenter penetration depth was the largest and amounted to 407 nm. In case of crystals of the last three intermetallics, values of indentation hardness *H_IT_* and indentation modulus *E_IT_* were definitely lower compared to values obtained for silicon crystals. Crystals of intermetallics rich in copper were characterized by lowest values of hardness *H_IT_* and modulus *E_IT_*, which is consistent with results reported in [55].

Based on published data concerning intermetallic phases Al_9_FeNi, Al_3_Ni, and Al_2_Cu, of which the first crystallizes at higher temperature and is characterized by higher value of the enthalpy and higher value *H_IT_* hardness and *E_IT_* modulus values, authors of [55] suggest that intermetallics development of which was connected with higher value of the enthalphy are characterized by stronger atomic bonds. It should be expected that the phase AlFeNi*M* (5) (*H_IT_* = 12,503–8575 MPa, *E_IT_* = 184–135 GPa) will crystallize before the phase Al_7_Cu_4_Ni (6) (*H_IT_* = 7682–5486 MPa, *E_IT_* = 157–115 GPa) and before the phase (7) Al_2_Cu (*H_IT_* = 6078–3918 MPa, *E_IT_* = 132–119 GPa).

The displacement of indenter face in the matrix during its loading and unloading is accompanied by plastic deformation of the material. A slight elastic deformation occurs in the course of unloading which can be explained by interaction of dislocations developing in the matrix material in the course of its plastic deformation. Such activity of dislocations is blocked by alloying elements reinforcing the matrix, which results in its plastic instability [55,60].

## 4. Conclusions

1. A new multicomponent hypereutectic silumin is developed for application in the process of manufacturing automobile cylinder cast-in liners.
2. The adopted chemical composition and conditions of solidification enabled crystallization of the intermetallic phase Al(Fe,Mn,*M*)Si, where *M* = Cr and V; the phase Al(Cr,V,*M*)Si where *M* = Mn, Fe, and Ti; the phase Al_4.5_FeSi; the phase AlFeNi*M* where *M* = Cu and Si; the phase Al_7_Cu_4_Ni; and phases Al_2_Cu and Mg_2_Si.
3. The addition of chromium, vanadium, and manganese partially blocked the development of AlFeSi phase, replacing iron in its composition and developing phases Al(Fe,Mn,*M*)Si and Al(Cr,V,*M*)Si characterized with *H*_IT_ and *E*_IT_ values higher than those observed for silicon crystals, which will be a feature of special importance for the service properties of cylinder liners.
4. It turned out that the intermetallic phase Al(Fe,Mn,*M*)Fe as well as the intermetallic Al(Cr,V,*M*)Si are characterized by lower susceptibility to the penetration of a diamond indenter compared to silicon crystals, and therefore better or comparable resistance to scratching with hard particles. This confirms usefulness of the newly developed silumin as a material suitable for automobile cylinder cast-in liners.

## Figures and Tables

**Table 1:** Chemical composition of hypereutectic aluminum-silicon alloy [13].

Element Content (wt.%) *											
**Si**	**Mn**	**Cu**	**Cr**	**Ni**	**V**	**Fe**	**Mg**	**Ti**	**B**	**P**	**Al**
31.32	0.53	1.44	0.58	1.15	0.45	0.54	1.33	0.041	0.0027	0.05	Bal.

* Alloy composition tested by Q4 Tasman emission spectrometer (Bruker, Kalkar, Germany).

## References

[1] Evans A., San Marchi C., Mortensen A. (2003). Metal Matrix Composites in Industry: An Introduction and a Survey.

[2] Köhler E., Niehues J., Sommer B. (2005). Vollaluminium–Zylinderkurbelgehäuse–Aluminium-Zylinderlauflächen. Giess. Prax..

[3] Wilson R. KS Aluminum Technologies AG is the master at making ALUSIL low pressure die casting engine blocks. *Automotive Industries*. https://www.ai-online.com/2003/10/supplier-technology-6/.

[4] Kainer K.U. (2006). Metal Matrix Composites. Custom-Made Materials for Automotive and Aerospace Engineering.

[5] Stocker P., Rückert F., Hummert K. (1997). Die neue Aluminium-Silizium-Zylinderlaufbahn-Technologie für Kurbelgehäuse aus Aluminiumdruckguss. MTZ Mot. Z..

[6] Krug P., Kennedy M., Foss J. New aluminum alloys for cylinder liner applications. Proceedings of the SAE World Congress Detroit.

[7] Scarlett M. (2004). Power trio: A pair of sixes and a 500 hp V-10 add up to a winning hand for BMW powertrain. Automot. Ind..

[8] Feikus F.J., Fischer A. (2001). Laserlegieren von Al-Zylinderkurbelgehäusen. VDI Ber..

[9] Schneider W. (2006). Highly stressed automotive engines made of aluminium. A challenges for founding technology and material development. Cast. Plant Technol..

[10] Kleine A., Koch H., Pohl J., Lindow H., Brinkmann R. (2009). Untersuchungen zu aluminiumbasierten Strukturverstärkungen für Zylinderkurbelgehäuse. Druckguss.

[11] Yamagata H., Kasprzak W., Aniolek M., Kurita H., Sokolowski J.H. (2008). The effect of average cooling rates on the microstructure of the Al-20% Si high pressure diecasting alloy used for monolithic cylinder blocks. J. Mater. Process. Technol..

[12] Yamagata H., Kurita H., Aniolek M., Kasprzak W., Sokolowski J.H. (2008). Thermal and metallographic characteristics of the Al-20% Si high-pressure die-casting alloy for monolithic cylinder blocks. J. Mater. Process. Technol..

[13] Orłowicz A.W., Mróz M.F., Tupaj M., Trytek A., Betlej J., Płoszaj F. (2012). A Silumin for Automobile Industry Castings and a Method for Shaping and Geometrical Surface Structure Increasing its Resistance to Abrasive Wear. Polish Patent.

[14] Horng J.H., Jiang D.S., Lui T.S., Chen L.H. (2000). The fracture behaviour of A356 alloys with different iron contents under resonant vibration. Int. J. Cast Met. Res..

[15] Warmuzek M., Sęk-Sas G., Lech Z. (2002). Ewolucja mikrostruktury w obecności metali przejściowych (Fe, Mn i Cr) w stopach Al-Si. Biul. Inst. Odlew..

[16] Manasijevic S., Radisa R., Markovic S., Acimovic-Pavlovic Z., Raic K. (2011). Thermal analysis and microscopic characterization of the piston alloy AlSi13Cu4Ni2Mg. Intermetallics.

[17] Abouei V., Saghafian H., Shabestari S.G., Zarghami M. (2010). Effect of Fe-rich intermetallics on the wear behavior of eutectic Al–Si piston alloy (LM13). Mater. Des..

[18] Saghafian H., Shabestari S.G., Ghadami S., Ghoncheh M.H. (2017). Effects of Iron, Manganese, and Cooling Rate on Microstructure and Dry Sliding Wear Behavior of LM13 Aluminum Alloy. Tribol. Trans..

[19] Bidmeshki C., Abouei V., Saghafianb H., Shabestari S.G., Noghani M.T. (2016). Effect of Mn addition on Fe-rich intermetallics morphology and dry sliding wear investigation of hypereutectic Al-17.5%Si alloys. J. Mater. Res. Technol..

[20] Yang Y., Zhong S.Y., Chen Z., Wang M., Ma N., Wang H. (2015). Effect of Cr content and heat-treatment on the high temperature strength of eutectic Al–Si alloys. J. Alloys Compd..

[21] Timelli G., Bonollo F. (2010). The influence of Cr content on the microstructure and mechanical properties of AlSi_9_Cu_3_(Fe) die-casting alloys. Mater. Sci. Eng. A.

[22] Uzun O., Kilicaslan M.F., Yılmaz F. (2014). Formation of novel flower-like silicon phases and evaluation of mechanical properties of hypereutectic melt-spun Al–20Si–5Fe alloys with addition of V. Mater. Sci. Eng. A.

[23] Liu H., Zhang H., Jiang F., Fu D. (2019). The intermetallic formation in the extruded AlSi20/8009 aluminum alloy during annealing treatment. Vacuum.

[24] Wu C., Lee S., Hsieh M., Lin J. (2010). Effects of Mg content on microstructure and mechanical properties of Al-14.5Si-4.5Cu Alloy. Metall. Mater. Trans. A.

[25] Zhang B., Zhang L., Wang Z., Gao A. (2020). Achievement of High Strength and Ductility in Al–Si–Cu–Mg Alloys by Intermediate Phase Optimization in As-Cast and Heat Treatment Conditions. Materials.

[26] Simensen C.J., Rolfsen T.-L. (1997). Production of π-AlMgSiFe crystals. Z. Met..

[27] Bolibruchova D., Macko J., Bruna M. (2014). Elimination of negative effect of Fe in secondary alloys AlSi6Cu4 (EN AC 45 000, A 319) by nickel. Arch. Metall. Mater..

[28] Meng F., Wu Y., Hu K., Li Y., Sun Q., Liu X. (2019). Evolution and Strengthening Effects of the Heat-Resistant Phases in Al–Si Piston Alloys with Different Fe/Ni Ratios. Materials.

[29] Murali S., Raman K.S., Murthy K.S.S. (1995). The formation of β-FeSiAl_5_ and Be-Fe phases in Al-7Si-0.3Mg alloy containing Be. Mater. Sci. Eng. A.

[30] Stolarz J., Madelaine-Dupuich O., Magnin T. (2001). Microstructural factors of low cycle fatigue damage in two phase Al-Si alloys. Mater. Sci. Eng..

[31] Abdulwahab M., Madugu I.A., Yaro S.A., Hassan S.B., Popoola A.P.I. (2011). Effects of multiple-step thermal ageing treatment on the hardness characteristics of A356.0-type Al-Si-Mg alloy. Mater. Des..

[32] Hernandez-Sandoval J., Garza-Elizondo G.H., Samuel A.M., Valtiierra S., Samuel F.H. (2014). The ambient and high temperature deformation behavior of Al-Si-Cu-Mg alloy with minor Ti, Zr, Ni additions. Mater. Des..

[33] Yanga Y., Yu K., Li Y., Zhao D., Liu X. (2012). Evolution of nickel-rich phases in Al-Si-Cu-Ni-Mg piston alloys with different Cu additions. Mater. Des..

[34] Yanga Y., Li Y., Wua W., Zhao D., Liu X. (2011). Effect of existing form of alloying elements on the microhardness of Al-Si-Cu-Ni-Mg piston alloy. Mater. Sci. Eng. A.

[35] Li Y., Yanga Y., Wua Y., Wang L., Liu X. (2010). Quantitative comparison of three Ni-containing phases to the elevated-temperature properties of Al–Si piston alloys. Mater. Sci. Eng. A.

[36] Li Y., Yanga Y., Wua Y., Wei Z., Liua X. (2011). Supportive strengthening role of Cr-rich phase on Al-Si multicomponent piston alloy at elevated temperature. Mater. Sci. Eng. A.

[37] Tupaj M., Orłowicz A.W., Mróz M., Trytek A. (2015). Materials Properties of Iron-rich Intermetallic Phase in a Multicomponent Aluminium-Silicon Alloy. Arch. Foundry Eng..

[38] Tupaj M., Orłowicz A.W., Mróz M., Trytek A., Markowska O. (2016). The Effect of Cooling Rate on Properties of Intermetallic Phase in a Complex Al-Si Alloy. Arch. Foundry Eng..

[39] PN-EN ISO 14577-1: 2015 (2013). Metallic Materials–Instrumented Indentation Test for Hardness and Materials Parameters–Part 1: Test Method.

[40] CSM Instruments (2010). Indentation Software Manual. Ver. R0.1.3.

[41] Rajabia M., Vahidia M., Simchia A., Davamia P. (2009). Effect of rapid solidification on the microstructure and mechanical properties of hot-pressed Al–20Si–5Fe alloys. Mater. Charact..

[42] Ünlü N., Genç A., Öveçoğlu M.L., Eruslu N., Froes F.H. (2001). Characterization investigations of melt-spun ternary Al–xSi–3.3Fe (x=10, 20 wt.%) alloys. J. Alloys Compd..

[43] Li G., Liao H., Suo X., Tang Y., Dixit U.S., Pavel Petrov P. (2018). Cr-induced morphology change of primary Mn-rich phase in Al-Si-Cu-Mn heat resistant aluminum alloys and its contribution to high temperature strength. Mater. Sci. Eng. A.

[44] Regulski K., Wilk-Kołodziejczyk D., Szymczak T., Gumienny G., Pirowski Z., Jaśkowiec K., Kluska-Nawarecka S. (2019). Data Mining Methods for Prediction of Multi-Component Al-Si Alloy Properties Based on Cooling Curves. J. Mater. Eng. Perform..

[45] Shabestari S.G. (2004). The effect of iron and manganese on the formation of intermetallic compounds in aluminum–silicon alloys. Mater. Sci. Eng. A.

[46] Sahoo K.L., Pathak B.N. (2009). Solidification behaviour, microstructure and mechanical properties of high Fe-containing Al–Si–V alloys. J. Mater. Process. Technol..

[47] Becker H., Leineweber A. (2018). Approximate icosahedral symmetry of α-Al(Fe,Mn,Cr)Si in electron backscatter diffraction analysis of a secondary Al-Si casting alloy. Mater. Charact..

[48] Schneider W. Highly stressed automotive engines of aluminium. Challenges for the casting technology and material development. Proceedings of the 66th World Foundry Congress.

[49] Heusler L., Feikus F.J., Otte M.O. (2001). Alloy and casting process optimization for engine block application. AFS Trans..

[50] Jang J., Lance M.J., Wen S., Tsui T.Y., Pharr G.M. (2005). Indentation-induced phase transformations in silicon: Influences of load, rate and indenter angle on the transformation behavior. Acta Mater..

[51] Gogotsi Y.C., Domnich V., Dub S.N., Kailer A., Nickel K.G. (2000). Cyclic nanoindentation and Raman microspectroscopy study of phase transformations in semiconductors. J. Mater. Res..

[52] Hu J.Z., Merkle L.D., Menoni C.S., Spain I.L. (1986). Crystal data for high-pressure phases of silicon. Phys. Rev. B Condens. Matter..

[53] Domnich V., Gogotsi Y. (2002). Phase transformations in silicon under contact loading. Rev. Adv. Mater. Sci..

[54] Bhattacharya S., Riahi A.R., Alpas A.T. (2009). Indentation-inducted subsurface damage in silicon particle of Al-Si alloys. Mater. Sci. Eng. A.

[55] Chen C.-L., Richter A., Thomson R.C. (2009). Mechanical properties of intermetallic phases in multi-component Al–Si alloys using nanoindentation. Intermetallics.

[56] Pietrowski S., Szymczak T. (2010). Crystallization, microstructure and mechanical properties of silumins with micro-addition of Cr, Mo, W and V. Arch. Foundry Eng..

[57] Bolibruchova D., Zihalov M. (2014). Vanadium influence on iron based intermetallic phases in AlSi6Cu4 alloy. Arch. Metall. Mater..

[58] Lin B., Li H., Xu R., Xiao H., Zhang W., Li S. (2019). Effects of Vanadium on Modification of Iron-Rich Intermetallics and Mechanical Properties in A356 Cast Alloys with 1.5 wt.% Fe. J. Mater. Eng. Perform..

[59] Zhan H., Hu B. (2018). Analyzing the microstructural evolution and hardening response of an Al-SiMg casting alloy with Cr addition. Mater. Charact..

[60] Chinh N.Q., Gubicza J., Kovacs Z., Lendvai J. (2004). Depth-sensing indentation tests in studying plastic instabilities. J. Mater. Res..

